# Antibiotic use among hospitalized patients in northern Nigeria: a multicenter point-prevalence survey

**DOI:** 10.1186/s12879-020-4815-4

**Published:** 2020-01-30

**Authors:** Usman Abubakar

**Affiliations:** Pharmacy Department, Ibrahim Badamasi Babangida (IBB) Specialist Hospital, Minna, Niger Nigeria

**Keywords:** Antibiotic use, Point-prevalence, Acute care hospitals, Northern Nigeria, Antibiotic stewardship, Redundant antibiotic combination

## Abstract

**Background:**

The evaluation of antibiotic use among hospitalized patients is a primary step required to design antibiotic stewardship intervention. There is paucity of data describing antibiotic use in hospitals across Northern Nigeria. This study evaluates the prevalence and indications for antibiotic use among inpatients in three acute care hospitals.

**Methods:**

A point-prevalence survey was conducted among patients in the wards before or at 8.00 a.m. on the day of the survey, using the point-prevalence survey of healthcare-associated infections and antimicrobial use in European acute care hospitals protocol. The survey was conducted between April and May 2019. The medical records of the patients were reviewed by a clinical pharmacist with the support of physicians and nurses.

**Results:**

Overall, 80.1% (257/321) of the patients used at least one antibiotic on the day of the survey. The prevalence of antibiotic use ranged from 72.9% in obstetrics and gynecology to 94.6% in pediatric medical specialty. Community acquired infections (38.7%) and surgical antibiotic prophylaxis (22.5%) were the most common indications. Surgical antibiotic prophylaxis was used or scheduled to be used for more than a day in all the cases. Metronidazole (30.5%), ciprofloxacin (17.1%), ceftriaxone (16.8%), amoxicillin-clavulanate (12.5%) and gentamicin (11.8%) were the most commonly prescribed antibiotics. Overall, broad spectrum antibiotics represented one-third of all the prescriptions. The change of initial antibiotic prescription was reported in one-third of the patients and the reasons include a switch to oral antibiotic (28.5%), escalation (4.5%) and de-escalation (3.6%). Of the 257 patients with an antibiotic prescription, 6.2% had redundant antibiotic combinations.

**Conclusion:**

The prevalence of antibiotic use was high with one in three prescriptions having a broad spectrum antibiotic. Prolonged use of surgical antibiotic prophylaxis and redundant antibiotic combination were observed. Antimicrobial stewardship interventions are recommended in order to reduce the use of antibiotics and promote appropriate antibiotics prescribing.

## Background

Globally, antimicrobial resistance has become a threat to public health security [1]. Antimicrobial resistance threatens the treatment of infections as well as advanced medical operations including surgery, dialysis, chemotherapy and organ transplant which rely on the availability of effective antibiotics. In addition, antimicrobial resistance is associated with morbidity, mortality and healthcare costs [2, 3]. The inappropriate use of antibiotic contributes to the emergence and spread of antimicrobial resistance [1]. Available evidence suggests that 20–50% of antibiotic prescription in the hospital is inappropriate [2]. Thus, antimicrobial stewardship is one of the strategies used to tackle the current antibiotic resistance crisis [1]. The antimicrobial stewardship program needs to be guided by data describing the prevalence and indications for antibiotic use in various clinical settings.

Point-prevalence survey is increasingly been used to monitor antibiotic prescribing patterns in acute and long-term healthcare facilities. This design has been found to be a valid and reliable method to measure and monitor antibiotic use in healthcare facilities [4]. It is often used to identify areas for quality improvement, to set benchmark, and to assess the impact of antimicrobial stewardship interventions [5]. Previous studies found that the prevalence of antibiotic use among hospitalized patients varied between 30.5% in Europe [6], 49.9% in the United States (US) [7], and 64.6% in Benin [8].

In Nigeria, studies showed that the prevalence of antibiotic use among hospitalized patients ranged from 62.4 to 78.2% [9–11]. Community acquired infection was the most common indication and third generation cephalosporin and nitro-imidazole were the most commonly prescribed antibiotics [9–11], however, most of the studies were conducted in a single tertiary healthcare center in the southern part of Nigeria. There is limited data describing the rate and indications for the use of antibiotics among hospitalized patients in Northern Nigeria. The objective of this study was to assess the prevalence of antibiotic use in secondary and tertiary acute care hospitals, and describe the indicators of quality antibiotic prescription including the documentation of reasons for antibiotic prescription and change of an initial antibiotic during current infection episode. Redundant antibiotics combination has been identified as a local problem in healthcare settings in the northern region [12]. The study also evaluated the prevalence, types and factors associated with redundant antibiotics combination in inpatient settings.

## Methods

### Study design

The point-prevalence survey was conducted in three acute care hospitals across two states in Northern Nigeria, between April and May 2019 using the point-prevalence survey of healthcare-associated infections and antimicrobial use in European acute care hospitals protocol version 5.3 [13]. The survey was conducted in one teaching (tertiary) and two general (secondary) hospitals in the region. The participating hospitals had approximately 850 beds at the time of the survey and provided acute care services including internal medicine, surgery, obstetrics and gynecology, pediatrics, neonatal, psychiatry, intensive care and ear, nose and throat care. The wards in each specialty in the participating hospitals were divided into male and female wards, the wards were further subdivided into ‘A’ and ‘B’ respectively. None of the wards of the participating hospital had more than 30 patients on admission at the time of the survey.

### Inclusion and exclusion criteria

Hospitalized patients in all the wards including pediatric emergency (where patients were often admitted for more than 24 h) and neonatal wards of the participating hospitals were considered for inclusion. However, those admitted to psychiatric, adult accident and emergency, and outpatient units were excluded. All hospitalized patients in the ward before or at 8.00 a.m. on the day of the survey were included, and those who were discharged from the wards before the time of the survey and daycare patients were excluded.

### Data collection

The data was collected using the European protocol for point-prevalence survey of healthcare-associated infections and antimicrobial use in European acute care hospitals version 5.3 [13]. In addition to the prevalence of antibiotic use, the current study collected data regarding the presence and types of redundant antibiotic therapy. The data was collected by a clinical pharmacist with expertise in antimicrobial stewardship and infectious diseases. The patients’ records were reviewed by the clinical pharmacist with the help of the attending physician, nurse and pharmacist.

The data collected includes: hospital type, ward specialty, patient’s demographic information and the presence of antibiotic prescription on the day or at the time of the survey. For those with at least one antibiotic prescription, the following information was collected for each antibiotic: name of drug, number of doses per day, strength of one dose, route of administration (oral, parenteral, inhalation), indication for prescription (community and hospital acquired infections, surgical prophylaxis, medical prophylaxis and unknown indication), diagnosis, documentation of reason for antibiotic prescription, date antibiotic was started, and whether antibiotic was changed during the current infection episode. For patients receiving surgical antibiotic prophylaxis, their medical records were reviewed to 24 h before the time of the survey to ascertain whether it was prescribed as a single dose, for less than 24 h or for more than 24 h. Medical records of those with potential redundant antibiotic combinations on the day of the survey were reviewed to 48 h prior to the time of the survey. All the patients in a single ward were surveyed on the same day. The data collection process lasted for 2 weeks in one of the participating hospitals. Ethics approval was obtained from the Human Research and Ethics Committee at the three hospitals with a request for waiver of patient informed consent (reference number: ABUTHZ/HREC/G23/2019, HMB/GHM/136/VOL.III/541 and MOH/ADM/744/VOL.1/716).

### Outcome measures

Redundant antibiotic therapy was defined as the prescription of two or more antibiotics with overlapping spectra of activity for 48 h or more [14, 15]. Dual anaerobic antibiotic prescriptions including oral metronidazole or vancomycin used for the treatment of *Clostridium difficile* infection or biliary tract infection were not classified as redundant [14]. Human immunodeficiency virus cases who received co-trimoxazole for the prevention or treatment of pneumocystis pneumonia in combination with another redundant antibiotic were excluded [15]. In addition, overlapping antibiotic spectra that involved erythromycin used in women with premature rupture of membrane was not included [15]. Those with redundant antibiotic therapy which was discontinued before the time of survey and those who received redundant antibiotic therapy for less than 48 h at the time of the survey were also excluded. Patient medical and nursing records and medication chart were reviewed by a clinical pharmacist, and potential redundant antibiotic therapy was discussed with the attending physician and/or nurse. The following antibiotics were classified as broad spectrum agents: piperacillin and beta-lactamase inhibitor, third- and fourth-generation cephalosporins, monobactams, carbapenems, fluoroquinolones, glycopeptides, polymyxins, daptomycin and oxazolidinones [6]. The World Health Organization’s Anatomical Therapeutic Chemical/Defined Daily Dose (ATC/DDD) index was used to classify the prescribed antibiotics into groups. The prevalence of antibiotic use and redundant antibiotic therapy was calculated as the percentage of patients who received one or more antibiotic and redundant antibiotic therapy on the day of the survey, respectively.

### Data analysis

The data was analyzed using Statistical Package for the Social Sciences (SPSS) version 23. The data was de-identified before analysis. Categorical data was reported as frequency with percentage while continuous data was presented as mean (with standard deviation) or median. Bivariate and multivariate regression analyses were used to determine the factors associated with redundant antibiotic therapy. Only variables that demonstrated statistical significance (*P* < 0.05) in the bivariate analysis were included in the multivariate model. *P* value less than 0.05 was considered as statistical significance.

## Results

A total of 321 inpatients were surveyed and the median age of the patients was 27 years with females representing approximately 58%. The majority of the surveyed patients were admitted to the medical unit (*n* = 87, 27.1%), obstetrics and gynecology (*n* = 70, 21.8%), surgical (*n* = 69, 21.5%) and pediatric medical unit (*n* = 37, 11.5%). Others were admitted to pediatric surgical (*n* = 30, 9.3%) and neonatal (*n* = 28, 8.7%) units. About one-third of the surveyed patients had surgery during the current admission.

### Prevalence of antibiotic use among hospitalized patients

Of the 321 patients surveyed, 80.1% had at least one antibiotic prescription on the day of the survey. The prevalence of antibiotic use was higher in pediatric medical specialty (94.6%) followed by neonatal (92.9%) and pediatric surgical (90.0%) specialties. Obstetrics and gynecology had the lowest prevalence (72.9%) (Table 1). Most of the patients (59.1%) had two antibiotic prescriptions while 33.5% had one prescription. Overall, 449 antibiotics were prescribed on the day of the survey and approximately 56% were parenteral prescriptions. The reason for antibiotic prescription was documented in the medical note in 75.8% of the cases.

**Table 1:** prevalence of antibiotic use among the specialty/ward

Variable	Frequency	Percentage
Prevalence of antibiotic use	257	80.1
Prevalence of antibiotic based on specialty		
Pediatric medical	35	94.6
Neonatal	26	92.9
Medical	64	73.6
Surgical	54	78.3
Obstetrics and gynecology	51	72.9
Pediatric surgical	27	90.0
Number of antibiotic prescription		
1	86	33.5
2	152	59.1
3	17	6.6
4	2	0.8
Routes of administration		
Parenteral	250	55.7
Oral	199	44.3
Indication for antibiotic prescription		
Community infection	174	38.7
Hospital infection	73	16.3
Medical prophylaxis	67	14.9
Surgical prophylaxis	101	22.5
Unknown indication	34	7.6

### Indications for antibiotic use

The most common indication for antibiotic prescription was community acquired infection (38.7%) followed by surgical antibiotic prophylaxis (22.5%) and hospital acquired infection (16.3%). Medical prophylaxis and unknown indication represented 14.9 and 7.6% of all the indications, respectively. Bloodstream infection (22.8), pneumonia (17.3%), skin and soft tissue infections (13.4%) and surgical site infections (11.0%) were the most common diagnoses with antibiotic prescription. Other diagnoses included gastro-intestinal infections (9.4%), intra-abdominal sepsis (7.9%), pyelonephritis (7.1%), obstetric or gynecological infection (3.9%) and central nervous system infection (3.1%). All the prescriptions for surgical antibiotic prophylaxis were prolonged beyond a day.

### Types of antibiotics prescribed

#### Classes of antibiotic prescribed

Overall, the most prescribed classes of antibiotic were nitroimidazoles (28.5%), third generation cephalosporins (18.9%), fluoroquinolones (13.6%), combinations of penicillins beta-lactamase inhibitors (10.5%) and aminoglycosides (8.5%). The proportion of broad spectrum antibiotics prescribed on the day of the survey was 32.9%. Broad spectrum antibiotic prescriptions constituted 33.9, 43.8, and 41.6% of antibiotics prescribed for community acquired infections, hospital acquired infections, and surgical antibiotic prophylaxis, respectively. Table 2 summarizes the classes of antibiotic used among the surveyed patients disaggregated based on the indications.

**Table 2:** classes of antibiotic prescribed among hospitalized patients based on the indications

Classes of antibiotic	Frequency (%)					
	Total	CI	HI	SP	MP	UI
Nitroimidazoles	128 (28.5)	46 (26.4)	23 (31.5)	34 (33.7)	13 (19.4)	12 (35.3)
Third generation cephalosporins	85 (18.9)	26 (14.9)	15 (20.5)	21 (20.8)	16 (23.9)	7 (20.6)
Fluoroquinolones	61 (13.6)	33 (19.0)	8 (11.0)	8 (7.9)	5 (7.5)	7 (20.6)
Penicillins – beta lactamase inhibitors	47 (10.5)	18 (10.3)	8 (11.0)	11 (10.9)	7 (10.4)	3 (8.8)
Aminoglycosides	38 (8.5)	13 (7.5)	10 (13.7)	4 (4.0)	10 (14.9)	1 (2.9)
Penicillins	38 (8.5)	11 (6.3)	4 (5.5)	8 (7.9)	12 (17.9)	3 (8.8)
Second generation cephalosporins	17 (3.8)	6 (3.4)	2 (2.7)	9 (8.9)	0 (0.0)	0 (0.0)
Lincosamides	14 (3.1)	9 (5.2)	1 (1.4)	3 (3.0)	1 (1.5)	0 (0.0)
Macrolides	8 (1.8)	6 (3.4)	0 (0.0)	1 (1.0)	1 (1.5)	0 (0.0)
Sulphonamides	4 (0.9)	2 (1.1)	1 (1.4)	0 (0.0)	1 (1.5)	0 (0.0)
Tetracyclines	4 (0.9)	1 (0.6)	0 (0.0)	2 (2.0)	0 (0.0)	1 (2.9)
Amphenicols	3 (0.7)	3 (1.7)	0 (0.0)	0 (0.0)	0 (0.0)	0 (0.0)
Carbapenems	1 (0.2)	0 (0.0)	1 (1.4)	0 (0.0)	0 (0.0)	0 (0.0)
Nitrofuran derivatives	1 (0.2)	0 (0.0)	0 (0.0)	0 (0.0)	1 (1.5)	0 (0.0)
Proportion of broad spectrum antibiotics	147 (32.7)	59 (33.9)	32 (43.8)	42 (41.6)	21 (31.3)	14 (41.2)

*CI* Community acquired infections, *HI* Hospital acquired infections, *SP* Surgical prophylaxis, *MP* Medical prophylaxis and *UI* Unknown indication

The classes of antibiotic prescribed on the day of the survey varied according to the indications. The five most commonly prescribed classes of antibiotic for community acquired infections include nitroimidazoles (26.4%), fluoroquinolones (19.0%), third generation cephalosporins (14.9%), combinations of penicillins beta-lactamase inhibitors (10.3%) and aminoglycosides (7.5%). In the case of hospital acquired infections, nitroimidazoles (31.5%), third generation cephalosporins (20.5%), aminoglycosides (13.7%), fluoroquinolones (11.0%) and combinations of penicillins beta-lactamase inhibitors (11.0%) were the most prescribed antibiotics. Of the 101 antibiotic prescriptions for surgical antibiotic prophylaxis, nitroimidazoles, third generation cephalosporins, combinations of penicillins beta-lactamase inhibitors, second generation cephalosporins and fluoroquinolones were the most prescribed and represented 33.7, 20.8, 10.9, 8.9 and 7.9% of all the prescriptions, respectively.

#### Antibiotics prescribed based on ward specialty

Antibiotic prescription varied among the specialties; ceftriaxone (28.8%), gentamicin (16.9) and metronidazole (13.6%) were the most commonly prescribed antibiotics in pediatric medical specialty. Among neonates, gentamicin (40.0%), ceftazidime (15.6%) and ampicillin-sulbactam (15.6%) were the most prescribed antibiotics. The three most common prescriptions in adult medical specialty were metronidazole (28.9%), ciprofloxacin (18.4%) and ceftriaxone (15.8%). In the surgical specialty, metronidazole (29.4%), ciprofloxacin (21.2%) and amoxicillin-clavulanate (15.3) were the most frequent antibiotic prescriptions while tinidazole (25.8%), cefixime (20.2%) and metronidazole (13.5%) were the most commonly prescribed antibiotics in the obstetrics and gynecology ward/specialty. Metronidazole, amoxicillin and gentamicin had the highest prescriptions and represented 36.8, 21.1 and 12.3%, of antibiotics prescribed in the pediatric surgical specialty, respectively. Table 3 shows the distribution of antibiotic prescriptions based on ward/specialty.

**Table 3:** Antibiotics prescribed among hospitalized patients disaggregated based on ward specialty

Antibiotic	Pediatric medical	Neonatal	Medical	Surgical	OBG	Pediatric surgical	Total
Metronidazole	8 (13.6)	0 (0.0)	33 (28.9)	25 (29.4)	12 (13.5)	21 (36.8)	99 (30.5)
Ciprofloxacin	3 (5.1)	0 (0.0)	21 (18.4)	18 (21.2)	7 (7.9)	6 (10.5)	55 (17.1)
Ceftriaxone	17 (28.8)	2 (4.4)	18 (15.8)	12 (14.1)	4 (4.5)	1 (1.8)	54 (16.8)
Amoxicillin-clavulanate	0 (0.0)	1 (2.2)	14 (12.3)	13 (15.3)	11 (12.4)	1 (1.8)	40 (12.5)
Gentamicin	10 (16.9)	18 (40.0)	2 (1.8)	0 (0.0)	1 (1.1)	7 (12.3)	38 (11.8)
Tinidazole	0 (0.0)	0 (0.0)	2 (1.8)	3 (3.5)	23 (25.8)	0 (0.0)	28 (8.7)
Cefixime	2 (3.4)	0 (0.0)	2 (1.8)	0 (0.0)	18 (20.2)	0 (0.0)	22 (6.9)
Amoxicillin	3 (5.1)	0 (0.0)	2 (1.8)	0 (0.0)	1 (1.1)	12 (21.1)	18 (5.6)
Cefuroxime	3 (5.1)	3 (6.7)	1 (0.9)	3 (3.5)	3 (3.4)	4 (7.0)	17 (5.3)
Clindamycin	0 (0.0)	1 (2.2)	4 (3.5)	7 (8.2)	1 (1.1)	1 (1.8)	14 (4.4)
Ampicillin-cloxacillin	4 (6.8)	4 (8.9)	0 (0.0)	1 (1.2)	2 (2.2)	0 (0.0)	11 (3.4)
Ceftazidime	0 (0.0)	7 (15.6)	2 (1.8)	0 (0.0)	0 (0.0)	0 (0.0)	9 (2.8)
Crystalline penicillin	4 (6.8)	2 (4.4)	1 (0.9)	0 (0.0)	0 (0.0)	1 (1.8)	8 (2.5)
Ampicillin-sulbactam	0 (0.0)	7 (15.6)	0 (0.0)	0 (0.0)	0 (0.0)	0 (0.0)	7 (2.2)
Azithromycin	1 (1.7)	0 (0.0)	5 (4.4)	0 (0.0)	0 (0.0)	0 (0.0)	6 (1.9)
Levofloxacin	0 (0.0)	0 (0.0)	2 (1.8)	2 (2.4)	0 (0.0)	2 (3.5)	6 (1.9)
Cotrimoxazole	1 (1.7)	0 (0.0)	2 (1.8)	0 (0.0)	1 (1.1)	0 (0.0)	4 (1.2)
Doxycycline	0 (0.0)	0 (0.0)	0 (0.0)	0 (0.0)	4 (4.5)	0 (0.0)	4 (1.2)
Chloramphenicol	2 (3.4)	0 (0.0)	1 (0.9)	0 (0.0)	0 (0.0)	0 (0.0)	3 (0.9)
Erythromycin	1 (1.7)	0 (0.0)	0 (0.0)	0 (0.0)	1 (1.1)	0 (0.0)	2 (0.6)
Ampicillin-floxacillin	0 (0.0)	0 (0.0)	1 (0.9)	0 (0.0)	0 (0.0)	0 (0.0)	1 (0.3)
Secnidazole	0 (0.0)	0 (0.0)	1 (0.9)	0 (0.0)	0 (0.0)	0 (0.0)	1 (0.3)
Meropenem	0 (0.0)	0 (0.0)	0 (0.0)	0 (0.0)	0 (0.0)	1 (1.8)	1 (0.3)
Nitrofurantoin	0 (0.0)	0 (0.0)	0 (0.0)	1 (1.2)	0 (0.0)	0 (0.0)	1 (0.3)

*OBG* Obstetrics and gynecology

#### Antibiotics prescribed based on indications

Metronidazole was the most commonly prescribed antibiotic for community acquired infections (23.6%), hospital acquired infections (20.5%), surgical antibiotic prophylaxis (20.8%) and unknown indication (32.4%). Other common antibiotic prescriptions include: ciprofloxacin (16.7%) and ceftriaxone (12.1%) for community acquired infections; gentamicin (13.7%) and ciprofloxacin (11.0%) for hospital acquired infection; and cefixime (12.9%) and amoxicillin-clavulanate (10.9%) for surgical antibiotic prophylaxis. Table 4 describes the distribution of antibiotics prescribed based on indications.

**Table 4:** Distribution of antibiotics prescribed based on indications

Antibiotic	CI	HI	SP3	MP	UI
Ciprofloxacin	29 (16.7)	8 (11.0)	7 (6.9)	4 (6.0)	7 (20.6)
Levofloxacin	4 (2.3)	0 (0.0)	1 (1.0)	1 (1.5)	0 (0.0)
Ceftriaxone	21 (12.1)	6 (8.2)	8 (7.9)	13 (19.4)	6 (17.6)
Cefuroxime	6 (3.4)	2 (2.7)	9 (8.9)	0 (0.0)	0 (0.0)
Cefixime	4 (2.3)	4 (5.5)	13 (12.9)	0 (0.0)	1 (2.9)
Ceftazidime	1 (0.6)	5 (6.8)	0 (0.0)	3 (4.5)	0 (0.0)
Metronidazole	41 (23.6)	15 (20.5)	21 (20.8)	11 (16.4)	11 (32.4)
Tinidazole	5 (2.9)	7 (9.6)	13 (12.9)	2 (3.0)	1 (2.9)
Secnidazole	0 (0.0)	1 (1.4)	0 (0.0)	0 (0.0)	0 (0.0)
Ampicillin-sulbactam	2 (1.1)	3 (4.1)	0 (0.0)	1 (1.5)	1 (2.9)
Amoxicillin-clavulanate	16 (9.2)	5 (6.8)	11 (10.9)	6 (9.0)	2 (5.9)
Amoxicillin	6 (3.4)	2 (2.7)	8 (7.9)	1 (1.5)	1 (2.9)
Ampicillin-cloxacillin	1 (0.6)	1 (1.4)	0 (0.0)	8 (11.9)	1 (2.9)
Ampicillin-floxacillin	0 (0.0)	0 (0.0)	0 (0.0)	0 (0.0)	1 (2.9)
Crystalline penicillin	4 (2.3)	1 (1.4)	0 (0.0)	3 (4.5)	0 (0.0)
Erythromycin	1 (0.6)	0 (0.0)	1 (1.0)	0 (0.0)	0 (0.0)
Azithromycin	5 (2.9)	0 (0.0)	0 (0.0)	1 (1.5)	0 (0.0)
Cotrimoxazole	2 (1.1)	1 (1.4)	0 (0.0)	1 (1.5)	0 (0.0)
Doxycycline	1 (0.6)	0 (0.0)	2 (2.0)	0 (0.0)	1 (2.9)
Chloramphenicol	3 (1.7)	0 (0.0)	0 (0.0)	0 (0.0)	0 (0.0)
Gentamicin	13 (7.5)	10 (13.7)	4 (4.0)	10 (14.9)	1 (2.9)
Clindamycin	9 (5.2)	1 (1.4)	3 (3.0)	1 (1.5)	0 (0.0)
Meropenem	0 (0.0)	1 (1.4)	0 (0.0)	0 (0.0)	0 (0.0)
Nitrofurantoin	0 (0.0)	0 (0.0)	0 (0.0)	1 (1.5)	0 (0.0)

*CI* Community acquired infections, *HI* Hospital acquired infections, *SP* Surgical prophylaxis, *MP* Medical prophylaxis and *UI* Unknown indication

### Change of antimicrobial during infection episode

Of the 446 prescriptions with data regarding change of an initial antibiotic, 170 (38.1%) were changed during the infection episode. The reasons for change of an initial antibiotic include switch from parenteral to oral therapy (*n* = 127, 28.5%), escalation (*n* = 20, 4.5%), de-escalation (*n* = 16, 3.6%) and unknown reason (*n* = 7, 1.6%). The initial antibiotic was not changed during the infection episode in 61.9% of the patients.

### Redundant antibiotic combinations

Twenty two cases of redundant antibiotic therapy were identified in 20 patients corresponding to a point-prevalence of 6.2% in all patients (and 11.7% among patients who received two or more antibiotics). The most common types of redundancy were dual anaerobic (77.3%) and dual beta-lactam (13.6%) therapy. Other types of redundancy include dual anti-*pseudomonal* (4.5%) and dual anti-*streptococcal* (4.5%). Redundancy was higher in oral (54.5%) compared to parenteral route (36.4%). Of the 22 redundant antibiotic therapy, 40.9% were prescribed for community acquired infections while 27.3 and 22.7% were used for hospital acquired infections and surgical antibiotic prophylaxis, respectively. Overall, the combination of amoxicillin-clavulanate with nitro-imidazoles (59%) was the highest type of redundant antibiotic therapy. Multivariate regression analysis showed that the number of antibiotics prescribed (AOR: 4.724, 95% CI: 1.595–13.991, *P* = 0.005) and oral antibiotics (AOR: 4.075, 95% CI: 1.340–12.390, *P* = 0.013) were independent predictors of redundant antibiotic therapy.

## Discussion

The study found that four in every five hospitalized patients received at least one antibiotic each day and approximately two-third of the antibiotics were used for treatment of community acquired infections and surgical prophylaxis. The prevalence of antibiotic use in the current study was consistent with a similar study conducted in South East Nigeria (78.6%) [10]. The prevalence of antibiotic use among hospitalized patients in Nigeria was higher than other African countries including Ghana (51.4%) [16] and Benin (64.6%) [8], Europe (30.5%) [6], and the United States (49.9%) [7]. The high rate of antibiotic use among hospitalized patients could be attributed to lack of a national antimicrobial guideline to promote rational use of antibiotics in hospital setting.

Approximately one-quarter of the antibiotics were prescribed for surgical antibiotic prophylaxis. Several studies have demonstrated that single dose antibiotic prophylaxis is as effective as multiple doses [17, 18]. Thus, it is recommended that surgical antibiotic prophylaxis should be discontinued within 24 h of completion of surgery in most cases [19]. However, all surgical antibiotic prophylaxis prescriptions in the current study were prolonged beyond 1 day. This implies that surgical antibiotic prophylaxis was overused, and confirmed the result of a previous study in Nigeria [12]. This observation highlights a potential target for antimicrobial stewardship intervention. A previous study conducted in Nigeria demonstrated that antimicrobial stewardship interventions significantly increased compliance with surgical antibiotic prophylaxis in obstetrics and gynecology procedures [20].

The study also found that majority (about 67%) of the patients who used antibiotic on the day of the survey had two or more prescriptions, similar to the result in Ghana [16]. However, the use of multiple antibiotic therapy among hospitalized patients was more than two times higher than Europe’s (29.4%) [6]. It is important to note that most of the surveyed patients had a single infectious disease diagnosis and high rate of multiple antibiotic prescription indicated the use of combination therapy. Although, combination antibiotic therapy will provide a broader spectra of activity and potential synergy, there is the lack of sufficient evidence to support its use in routine treatment of infections [21]. The frequent use of combination therapy in our setting could be attributed to the desire of the physician to provide coverage against all the possible infective pathogens owing to inadequate diagnostic infrastructure. Combination antibiotic therapy represents another potential area for the implementation of antimicrobial stewardship intervention.

Overall, nitroimidazoles, third generation cephalosporins and fluoroquinolones were the most commonly prescribed antibiotic groups. This was similar to previous findings in Southern Nigeria [10, 11]. In addition, metronidazole, ciprofloxacin, ceftriaxone, and amoxicillin clavulanate were the most used antibiotics among the surveyed patients. Again, this was consistent with the studies conducted in the southern parts of Nigeria [10, 11], but was inconsistent with the results in neighboring African countries including Ghana and Benin [8, 16]. These inconsistencies could be explained by differences in antimicrobial resistance patterns and empirical antimicrobial treatment recommendations between the countries. The study also revealed that broad spectrum antibiotics represented about one-third of all antibiotic prescriptions. Although, this was lower than the rate reported in Europe (about 40%) [6], the use of third generation cephalosporin and fluoroquinolone use in Nigeria needs to be monitored because these classes of antibiotic increase the risk of *Clostridium difficile* infection [22] and the emergence and spread of extended spectrum beta-lactamase (ESBL) infections [23]. The excessive use of fluoroquinolone among hospitalized patients could explain the high rate of resistance to the antibiotics among clinical isolates in Nigeria [24]. Therefore, antimicrobial stewardship intervention is required to promote appropriate use of broad spectrum antibiotics in the hospital setting.

The review of initial antibiotic prescription is an important antimicrobial stewardship strategy used to reduce overuse of antibiotics, particularly broad spectrum antibiotics. The current study found that review of initial antibiotic prescription was common as demonstrated by the change of about 40% of all the prescriptions. This confirmed the result of a previous study in Nigeria [10]. In the current study, switch to oral antibiotic and de-escalation were reported in about one-third of the prescriptions indicating frequent review of prescription based on clinical and microbiological parameters. Although this was higher than the result of the European survey [6], there is still room for further improvement. Reason for antibiotic use was not documented in about one-quarter of the prescriptions and this was higher than the rate reported in Europe and the US [6, 7]. Thus, more effort is required to encourage physicians to document the reason for antibiotic prescription in patient’s case note.

Another important finding of the current study was the relatively high rate of redundant antibiotic combination, approximately 12% of patients with two or more antibiotics had redundant antibiotic prescription with redundant anaerobic combinations, particularly the combination of amoxicillin-clavulanate with nitro-imidazoles, been the most common. These observations were consistent with previous studies conducted in the United States for prevalence [15] and type of redundancy [25], respectively. The reason for redundant antibiotic combinations in the current study is not clear. Previous studies indicated that the lack of knowledge about the spectrum of antibiotic activity, multiple antibiotic prescribers, gaps in antibiotic prescribing and distribution system and the desire to provide broader spectrum of activity are possible reasons for redundant antibiotic combination [14, 15, 25]. Reducing redundant antibiotic combinations should be considered as a priority for antimicrobial stewardship program in Nigeria. It is considered as an easy target for antimicrobial stewardship intervention [14].

Overall, the current study highlights the need for the establishment of antimicrobial stewardship program in Nigerian hospitals considering the high rate of antibiotic use, prolonged use of surgical prophylaxis, considerably high proportion of broad spectrum antibiotic, and use of redundant antibiotic combinations. A recent study revealed that only few hospitals had a formal antimicrobial stewardship team [26]. Lack of training in antimicrobial stewardship and lack of support from hospital administrators were identified as major barriers to participation in the program by hospital pharmacists. Public health authorities in Nigeria should encourage and support hospitals to establish antimicrobial stewardship program to promote rational use of antibiotics.

The current study has a number of limitations and thus, should be interpreted with caution. First, this is a 1 day cross sectional survey (point-prevalence survey) and it is not the gold standard for assessing antibiotic use. However, point-prevalence survey design is considered as a valid method for measuring antibiotic use in healthcare facilities. Secondly, the survey was conducted in only three hospitals, thus, the findings are not generalizable. However, the findings were comparable with previous studies conducted in the southern parts of Nigeria. Thirdly, the current study did not evaluate the appropriateness of antibiotic prescription among the patients. And fourthly, the sample size of the study was small. There is a need for a more elaborate study that will encapsulate the concerns highlighted above.

## Conclusion

The prevalence of antibiotic use among hospitalized patients was relatively high with broad spectrum antibiotics constituting about one-third of all antibiotic prescriptions. Antibiotics were commonly used for community acquired infections and surgical antibiotic prophylaxis in which the duration was prolonged beyond 1 day in all cases. Metronidazole, ciprofloxacin and ceftriaxone were the most commonly prescribed antibiotics. Approximately, one in 15 antibiotic prescriptions had redundant antibiotic combination. Antimicrobial stewardship interventions are strongly recommended to reduce the high rate of antibiotic prescription and promote appropriate use of antibiotics in Nigerian hospitals.

## Data Availability

All data generated or analyzed during this study are included in this published article.

## Abbreviations

CI: Community acquired infections
HI: Hospital acquired infections
MP: Medical prophylaxis
SP: Surgical prophylaxis
UI: Unknown indication
US: United States
